# Morphological transformation of gold nanoparticles on graphene oxide: effects of capping ligands and surface interactions

**DOI:** 10.1186/s40580-018-0171-0

**Published:** 2019-01-08

**Authors:** Hanqing Pan, Serena Low, Nisala Weerasuriya, Bingli Wang, Young-Seok Shon

**Affiliations:** Department of Chemistry and Biochemistry, California State University, Long Beach, 1250 Bellflower Blvd, Long Beach, CA 90840 USA

**Keywords:** Nanoparticles, Nanorods, Graphene oxide, Stability, Morphology, Gold

## Abstract

This article presents the influence of capping ligand and surface interaction types on the coarsening or reshaping behavior of surface-immobilized gold nanoparticles with different core size and shape. The morphological transformation of gold nanoparticles and nanorods on graphene oxide upon heating at temperatures ranging from 50 to 200 °C was investigated. The aggregation and coarsening behaviors of spherical nanoparticles on graphene oxide were slightly affected by the core size of nanoparticles (~ 1, 3, and 10 nm). The comparison of two different surface ligands revealed that glutathione ligands provide much better protection than cetyltrimethylammonium bromide ligands against the morphological transformation of nanoparticles. In addition, the evaluation of surface binding interactions indicated that the attachment of nanoparticles and nanorods onto graphene oxide with additional thiol functional groups could improve the immobilization of particles and therefore decelerate coarsening and reshaping of nanoparticle and nanorods.

**Electronic supplementary material:**

The online version of this article (10.1186/s40580-018-0171-0) contains supplementary material, which is available to authorized users.

## Introduction

Metal nanoparticles are a class of functional materials with unique physical and chemical properties, which are closely related to their size, shape, composition, and structure [1, 2]. Especially, gold nanoparticles (AuNP) and gold nanorods (AuNR) have gained a lot of attention because of their applications in catalysis, electronics, and sensors [2–11]. They have also exhibited many attractive features such as strong plasmonic activity, facile ligand functionalization, water solubility, and excellent biocompatibility, which have made them good candidates for biomedical applications [6, 7, 9–17]. Deposition of AuNPs on solid supports has often been necessary for various reasons including enhanced stability and synergistic activity [2, 5, 10, 11, 14]. Therefore, the preparation of nanoparticle hybrids with the appropriate support materials and understanding the influence of supports to nanoparticles are critical to the expansion of practical applications of AuNPs [18, 19].

Since AuNPs anchored on graphene families could exhibit interesting catalytic, electrical and optical activities [2, 5, 8, 10, 11, 20], understanding the nature and extent of morphological transformation of gold nanoparticle-graphene oxide (AuNP-GO) hybrids could maximize their potentials for many practical applications [18–23]. Previous studies from our research group have shown that the presence of GO supports negatively affects the stability of AuNPs and AuNRs causing their core to undergo size and/or shape evolutions more easily [24]. The disruption and stripping of the protecting organic ligands by GO through strong electrostatic interactions were proposed as a main mechanism of nanoparticle destabilization on GO.

The gradual changes in size and shape of nanoparticles, when they are exposed to heat or light irradiation [21, 22, 25–27], could seriously impede their long-term technological advancements for device and catalysis applications. Therefore, further understanding of various factors determining morphological transformations of nanoparticles is required for developing new strategies for improving their overall stability and performance. In this study, AuNPs with different core sizes are assembled on the surface of GO to study their heat-induced coarsening behavior. AuNPs with different surface capping ligands (glutathione and cetyltrimethylammonium bromide (CTAB)) on GO was also prepared to understand the relationship between the stability of AuNPs and the type of surface ligand-nanoparticle interactions. The chemical interaction or bond formation between GO supports and ligand-capped AuNPs was also controlled by using thiol-functionalized GO (tGO) to see the influence of additional metal-sulfur bond against the morphological transformation of AuNP and AuNR. Various AuNP-GO and AuNR-GO hybrid materials are annealed from 50 to 200 °C to examine the influence of various structural components.

## Experimentals

Detailed information regarding materials, synthetic procedures, and characterizations (^1^H NMR, FT-IR, and TGA) for glutathione-capped gold nanoparticles (gAuNP) [28–31], cetyltrimethylammonium bromide (CTAB)-capped gold nanoparticles (cAuNP) [9], and CTAB-capped gold nanorods (cAuNR) [9], and instrumental methods used to conduct the research presented in this article are provided in the previous publications and Additional file 1 [15, 24, 29].

### Preparation of thiol-functionalized graphene oxide (tGO)

Thiol-functionalized graphene oxide was made by following the published procedure [22, 30, 32]. First, 5 mL of 0.4 M EDC (1-ethyl-3-(3-dimethylaminopropyl)carbodiimide), 5 mL of 0.1 M NHS (N-hydroxysuccinimide), and 5 mL 0.1 M l-cysteine were prepared. A 0.5 mL solution of concentrated hydrochloric acid was added to the l-cysteine solution to dissolve the l-cysteine crystals. Solutions of EDC, NHS, and l-cysteine were added to 8 mL of 4 mg/mL graphene oxide suspension in the order mentioned. As soon as the EDC/NHS/l-cysteine solutions were added, graphene oxide begins to separate from solution due to the decrease in polarity of GO with the incorporation of thiol functional groups from l-cysteine. The mixture is left sitting for 10 min, then centrifuged. The supernatant is discarded, and the precipitate was washed with nanopure water and centrifuged to precipitate the tGO. This process was repeated twice. The final product was left to dry in a vacuum overnight. IR spectra of isolated tGO showed the presence of S–H stretching bands at ~ 2565 cm^−1^.

### Preparation of gold nanoparticle-graphene oxide (AuNP-GO) and nanorod-graphene oxide (AuNR-GO) hybrids

Graphene oxide solution was made by dispersing GO flakes in nanopure water. GO flakes were not readily dispersed in water, hence the mixture was sonicated for 30 min until the GO formed a homogeneous dispersion. The concentration of GO solution was 1 mg/mL. The GO dispersion in water exhibited long-term stability. The gAuNP of size ~ 1, 3, and 10 nm, cAuNP, and cAuNR were bonded to GO as follows: Gold nanorods/nanoparticles were dissolved in water (~ 1 mg/mL) forming homogeneous solutions. The solutions of GO and gold nanorods/nanoparticles were mixed at a 1:1 ratio (v/v: 1 mL GO solution to 1 mL gold nanorod/nanoparticle solution) for 24 h.

To prepare the hybrids of tGO with AuNP and AuNR, the dried tGO was re-dispersed in nanopure water and the aqueous solutions of AuNP and AuNR were added to this solution. The mixture was then stirred at room temperature for 24 h to allow the formation of metal-sulfur bonds, so that the nanoparticles and nanorods can be covalently anchored to graphene oxide. The final hybrid product is then centrifuged, and the supernatant was discarded. The resulting precipitate was stored in a vacuum desiccator for at least 72 h to remove all solvents.

### Heat-treatment of AuNP-GO and AuNR-GO hybrids

The hybrids were heated from 50 to 200 °C, at 50 °C increments using a Barnstead Thermolyne 1300 Furnace. Each sample was heated for 1 h in a glass vial in air. Solvents were removed to dryness before the particle samples were heated in powder form.

## Results and discussion

### Gold nanoparticles with different sizes and stabilizing ligands

**Fig. 1:**
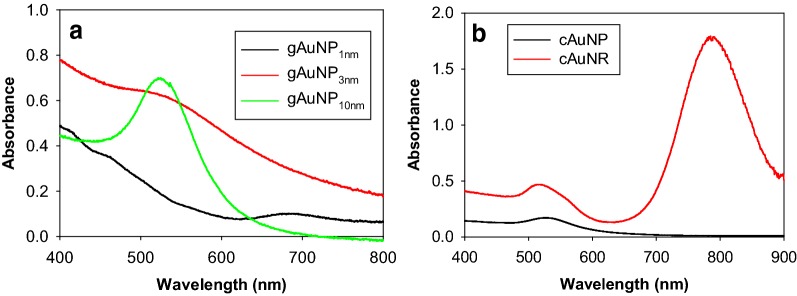
UV-vis spectra of **a** gAuNP with core sizes of ~ 1, 3, and 10 nm and **b** cAuNP and cAuNR

UV–vis spectra of gAuNP with different core sizes are shown in Fig. 1a, which confirmed the optical properties of gold nanoparticles corresponding to their expected sizes. The UV–vis spectrum of gAuNP_10nm_ exhibits strong surface plasmon (SP) bands at ~ 520 nm, while that of gAuNP_3nm_ show less prominent SP bands at the similar wavelength [29]. The UV–vis spectrum of small and monodispersed gAuNP_1nm_ does not possess plasmon bands, but have three absorption bands at 680, 450, and 410 nm, which are in a good agreement with previous reports published on the characteristics of Au_25_(GS)_18_ [30, 31]. Both cAuNP_3nm_ and cAuNR were prepared following a published method by Castellana et al. [9]. The UV–vis spectrum of cAuNP_3nm_ showing small SP bands at ~ 520 nm is quite similar to that of gAuNP_3nm_ (Fig. 1b). The characteristic plasmon bands at 520 nm (transverse) and 780 nm (longitudinal) shown in the UV–vis spectrum of cAuNR confirmed the successful preparation of monodisperse gold nanorods.

**Fig. 2:**
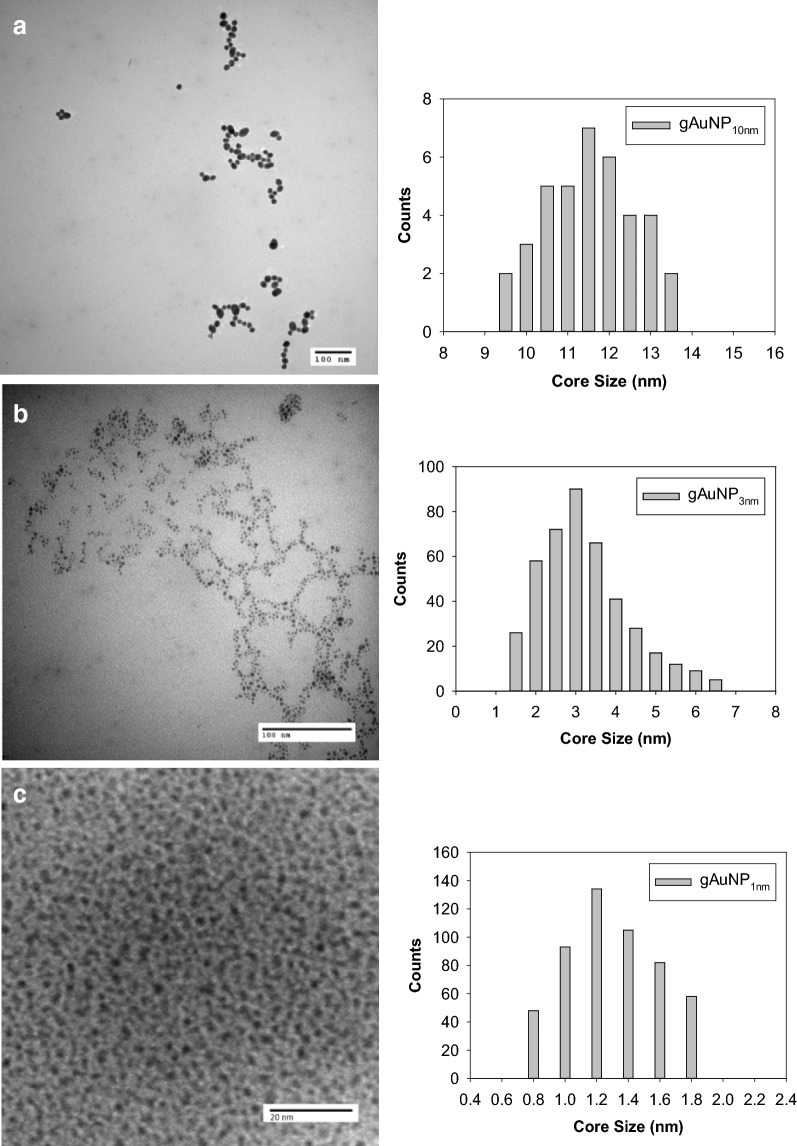
TEM images and histograms of **a** gAuNP_10nm_, **b** gAuNP_3nm_, and **c** gAuNP_1nm_

TEM images and their respective histograms showing size distribution of gAuNP are shown in Fig. 2. TEM images of both gAuNP_10nm_ and gAuNP_3nm_ (Fig. 2a, b), respectively) show that the nanoparticles are monodispersed and not aggregated. Histograms show that the majority of gAuNP_3nm_ are indeed populated around ~ 3 nm and the gAuNP_10nm_ have an average size of ~ 11.5 nm. TEM image (Fig. 2c) of the small and monodispersed gAuNP_1nm_ corresponds well with UV–vis results, being that they are too small to possess plasmon bands. The histogram of gAuNP_1nm_ shows that the core size of the nanoparticles is highly populated at ~ 1.2 nm.

**Fig. 3:**
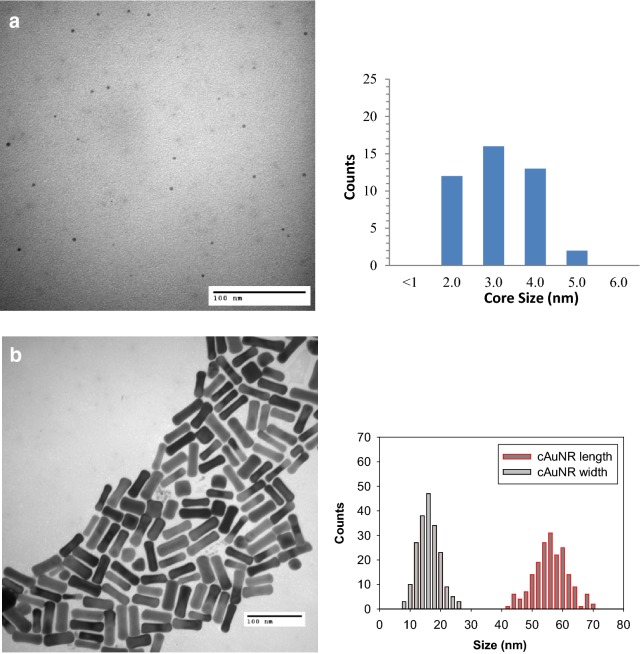
TEM images and histograms of **a** cAuNP_3nm_ and **b** cAuNR

TEM images of cAuNP_3nm_ and cAuNR (Fig. 3) show that the cAuNP_3nm_ have spherical shape with average core size of ~ 2.7 nm and the cAuNR are highly ordered, uniform in size and shape, and show no evidence of coarsening. Histograms for cAuNR show size distributions for both length and width with the majority of nanorods being ~ 55 nm in length and ~ 15 nm in width (an aspect ratio of 3.5:1). The first column of Table 1 summarizes the average core size and their respective standard deviations of the gAuNP, cAuNP, and cAuNR.

**Table 1:** Core size evolution of gold nanoparticles and nanorods (nanometers)

	Free standing	Supported on GO (or tGO)	Heated at 50 °C	Heated at 100 °C	Heated at 150 °C	Heated at 200 °C
gAuNP_10nm_	11.1 ± 1.2	10.1 ± 6.9	10.9 ± 4.8	11.5 ± 6.6	20.2 ± 8.6	22.1 ± 6.2
gAuNP_3nm_	3.0 ± 1.4	3.0 ± 0.9^a^ 2.9 ± 1.1 (on tGO)	3.1 ± 1.8^a^ 3.1 ± 1.1 (on tGO)	3.3 ± 1.6^a^ 3.3 ± 1.6 (on tGO)	7.0 ± 3.6^a^ 5.1 ± 2.0 (on tGO)	10.1 ± 5.4^a^ 7.6 ± 3.8 (on tGO)
gAuNP_1nm_	1.2 ± 0.3	1.3 ± 0.4	1.4 ± 0.5	1.4 ± 0.7	4.7 ± 0.8	5.3 ± 1.6
cAuNP_3nm_	2.7 ± 1.1	3.6 ± 1.6	10.0 ± 6.0	13.7 ± 10.5	68.5 ± 20.1	–
cAuNR	53.6 ± 8.2 15.1 ± 3.6	53.8 ± 13.2^a^ 17.3 ± 6.3^a^ 54.4 ± 7.1 14.8 ± 2.3 (on tGO)	89.4 ± 52.3^a^ n.a^a^ 52.4 ± 6.7 12.9 ± 2.2 (on tGO)	– –	– –	– –

^a^ The data are from the previous publication [24]

### Gold nanoparticles and nanorods on graphene oxide

**Fig. 4:**
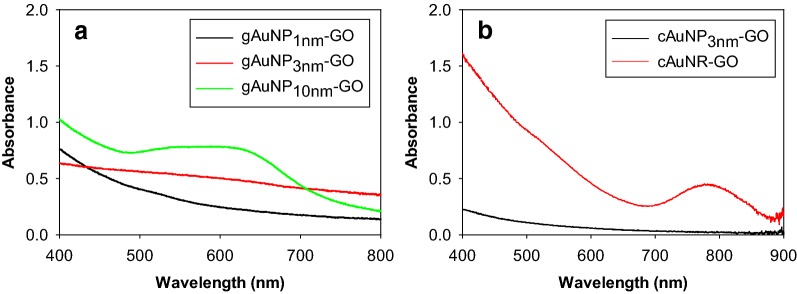
UV-vis spectra of **a** gAuNP_10nm_-GO, gAuNP_3nm_-GO, and gAuNP_1nm_-GO and **b** cAuNP_3nm_-GO and cAuNR-GO

The gAuNP with different sizes were attached to the surface of GO via non-covalent interactions such as hydrogen bonding and electrostatic interactions between COOH/OH groups of GO and COO^−^/NH_2_ groups of glutathione ligands on gAuNP. The cAuNP and cAuNR were self-assembled onto the surface of GO mostly via electrostatic interactions of R_4_N^+^ groups of bilayer CTAB ligands with the COO^−^ groups of GO. UV–vis spectra of gAuNP-GO with different core sizes, cAuNP_3nm_-GO, and cAuNR-GO are shown in Fig. 4. When gAuNP_10nm_ are assembled onto GO, the SP bands of gold broadens and red-shifts, which are consistent with the results reported by Huang et al. [33]. It has been shown that such a change in the plasmon band of the large gold nanoparticles in the GO hybrids indicate the significant formation of AuNP aggregates on the GO sheets [34, 35]. After the adsorption of gAuNP_3nm_ on GO, the SP bands of gold at ~ 520 nm mostly disappear as shown in the UV–vis spectrum. In our previous work, we found that this optical change is not associated with the core size change of AuNP after adsorption [24]. For the UV–vis spectrum of gAuNP_1nm_, three characteristic absorption bands at 680, 450, and 410 nm completely disappeared after the assembly onto GO exhibiting only an exponential decay in absorbance with a decrease in energy. The spectra feature now resembles that of 1.5–2 nm AuNP which do not possess any significant adsorption and SP bands in the UV–vis spectra. Recently, Ghosh et al. [36] have shown that Au_25_SR_18_ clusters undergo core size evolution to Au_135_ clusters after the deposition on GO in THF. This result indicated that the disappearance of three absorption bands after adsorption onto GO and an appearance of small hints of SP bands at ~ 520 nm in UV–vis spectrum of the gAuNP_1nm_-GO hybrids was likely caused by the core size evolution of small and less stable gold cluster core to gold nanoparticles with core size of > 1.4 nm.

When cAuNP_3nm_ and cAuNR are deposited on GO, the SP bands at ~ 520 nm almost completely disappear and the longitudinal plasmon band of cAuNR decreases in intensity (Fig. 4b), which is another piece of evidence for electronic and optical interference of GO for plasmonic nanostructures. Such changes in the plasmon response of cAuNR adsorbed on GO have also been observed by others [37, 38]. The absence of any shift for the longitudinal plasmon band of cAuNR indicated, however, that the aspect ratio of cAuNR mostly remained the same.

Additional file 1: Figure S1 shows TEM images of various gAuNP-GO hybrid materials and their respective histograms showing size distribution. GO sheets are apparent in all the TEM images as darker regions. The actual particle core size of gAuNP_10nm_-GO is estimated to be ~ 10 nm, which is similar to the size of free standing gAuNP_10nm_ (Table 1). However, the images showed many particles to be positioned closely together making an accurate histogram analysis a bit more difficult. These apparent formations of nanoparticle aggregates must be the main reason for the large red shift of the SP bands of gAuNP_10nm_ after GO adsorption. TEM image and histogram results agreed that the gAuNP_3nm_-GO have relatively well dispersed nanoparticles with the average core size near ~ 3 nm. The histogram results indicated gAuNP_3nm_ are relatively stable after GO immobilization showing no evidence of core size evolution. For gAuNP_1nm_-GO hybrids, TEM images and histogram results indicated there is a slight increase in average core size of gAuNP_1nm_ after adsorption onto GO. This size change corresponded well with the UV–vis results of gAuNP_1nm_-GO and support the previous discovery reported by Ghosh et al. [36] regarding the size evolution of Au_25_ clusters when in contact with graphene substrate.

TEM results shown in Additional file 1: Figure S2 indicated that cAuNP_3nm_ and cAuNR are immobilized on GO without any aggregation or reshaping maintaining a similar core size and an aspect ratio of over 3 (~ 54 nm in length and ~ 17 nm in width), respectively. The TEM results agreed relatively well with their corresponding UV–vis spectra of their original sizes and shapes shown in Fig. 4b. The dampening of the plasmon bands of cAuNP_3nm_ and cAuNR at ~ 520 nm was clearly not a result of any significant morphological size or shape changes.

When comparing the core sizes of the nanoparticles before and after their assembly onto GO, it is clear that the use of pre-formed nanoparticles mostly affords the retention of core size and dispersity of assembled nanoparticles without any significant change. However, very small clusters (~ 1 nm) and large colloidal nanoparticles (> 10 nm) that usually exhibit low stability alone in solution phase are also susceptible to a slight change in their morphologies after immobilization on GO. This result is confirmed by TEM analysis and the changes in their optical activity. Gold nanorods immobilized on GO avoided extensive aggregations suggesting the presence of CTAB bilayer ligands on their surfaces.

### Effects of core size on coarsening of gAuNP on GO

Our previous work has shown that the presence of GO facilitates the coarsening of gAuNP_3nm_ [24]. As shown in Table 1, large increases in the average core size of gAuNP_3nm_ on GO took place at heating temperatures between 100 and 150 °C. The transition, however, took place between 150 and 200 °C for free standing gAuNP_3nm_ [24]. The hypothesis was that the GO, being a flexible material, can bend and wrap around the nanoparticles and chemically interact with the ligands due to the presence of many oxygen-containing functional groups. The ligands on AuNP are then pulled away from the nanoparticle and spilled over to GO during heat treatment facilitating the coarsening of the nanoparticles.

Heat treatments of gAuNP_10nm_-GO and gAuNP_1nm_-GO were performed from 50 to 200 °C at 50 °C increments to see the effects of particle size on the heat-induced coarsening behavior of gAuNP. UV–vis spectra of gAuNP_10nm_-GO showed the decreased intensity and significant broadening of SP bands after the heat treatments of the hybrids at 100 °C, which is lower than the temperature required for gAuNP_3nm_-GO (Additional file 1: Figure S3). The transitions continued with heating at increased temperatures and quickly reached the status of film-like insoluble materials showing only small scattering effects [39–41]. This fast transition would likely be caused by the formation of particle aggregations upon the adsorption of gAuNP_10nm_ on GO.

**Fig. 5:**
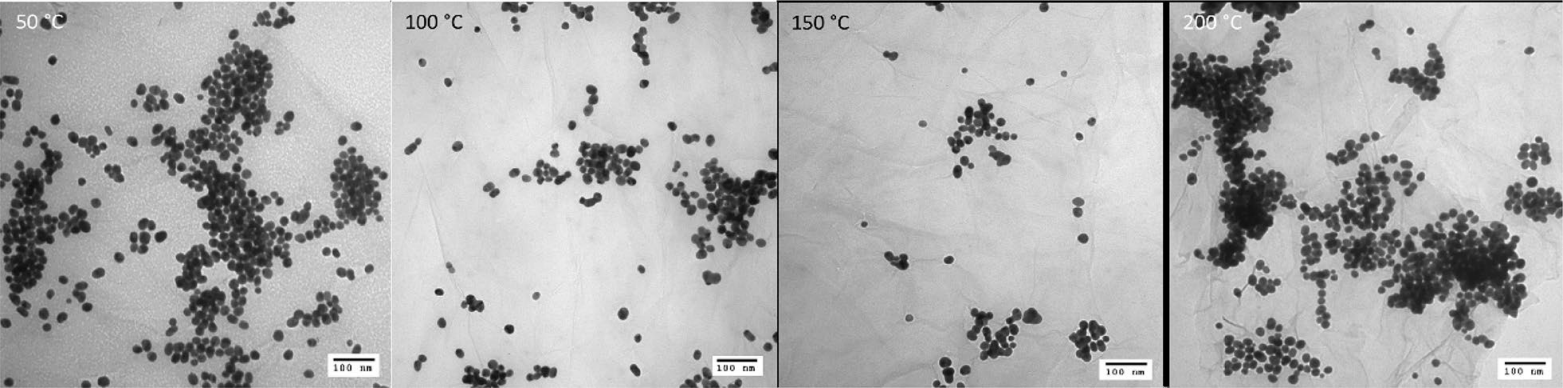
TEM images (scale bars = 100 nm) and size distribution of gAuNP_10nm_-GO heated at 50, 100, 150, and 200 °C

TEM results clearly indicated that the gAuNP_10nm_ on GO undergo coarsening at the heating temperature at or above 100 °C (Fig. 5). After heat treatment at 100 °C (Fig. 8, 100 °C), particles are more aggregated with a few particles slightly merging together. After the heat treatment at 150 and 200 °C (Fig. 8, 150 °C, 200 °C), it is clearly visible from TEM images that the particles are coarsening. Table 1 summarizes the changes in the particle size of the heated gAuNP_10nm_ on GO. A large increase in the average core size of gAuNP_10nm_ was observed from 100 to 150 °C suggesting the overall transitions are similar to that of gAuNP_3nm_ on GO and relatively in good agreement with both UV–vis results shown in Additional file 1: Figure S3 and those of gAuNP_3nm_-GO hybrids shown previously (Table 1).

UV–vis spectra of gAuNP_1nm_-GO (Additional file 1: Figure S4) show that the transition is gradually taking place throughout the heating temperature between 50 and 200 °C with decreasing intensity of spectra. The film-like spectral feature has appeared after heat treatments at 200 °C. This overall transition after heat treatment resembles quite similarly to that of gAuNP_10nm_-GO as shown above. The presence of small SP bands of gold is apparent for gAuNP_1nm_-GO after heating at 50 °C, which indicates some aggregation and coarsening of nanoparticles to slightly larger nanoparticles (~ 1.5–2.5 nm). The absence of any strong SP bands after heating at high temperature, however, indicates that the significant coarsening of gAuNP_1nm_ on GO to larger (> 3 nm) particles did not take place.

TEM images of the heated gAuNP_1nm_-GO are shown in Additional file 1: Figure S5 and the changes in the average core size and distribution are summarized in Table 1. The results suggested that the more rapid transition is observed at temperature below 150 °C for the gAuNP_1nm_-GO compared to other gAuNP on GO. The gAuNP_1nm_-GO began to reach the extensive coarsening stage and became mostly insoluble during the heat treatment at 200 °C. These results also corresponded well with the data obtained from UV–vis spectra.

### Effects of capping ligands for coarsening of gold nanoparticles on graphene oxide (gAuNP_3nm_-GO vs cAuNP_3nm_-GO)

**Fig. 6:**
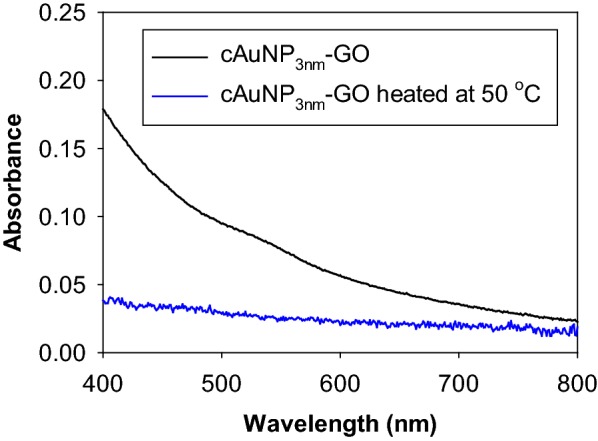
UV-vis spectra of cAuNP_3nm_-GO before and after heat treatment at 50 °C

**Fig. 7:**
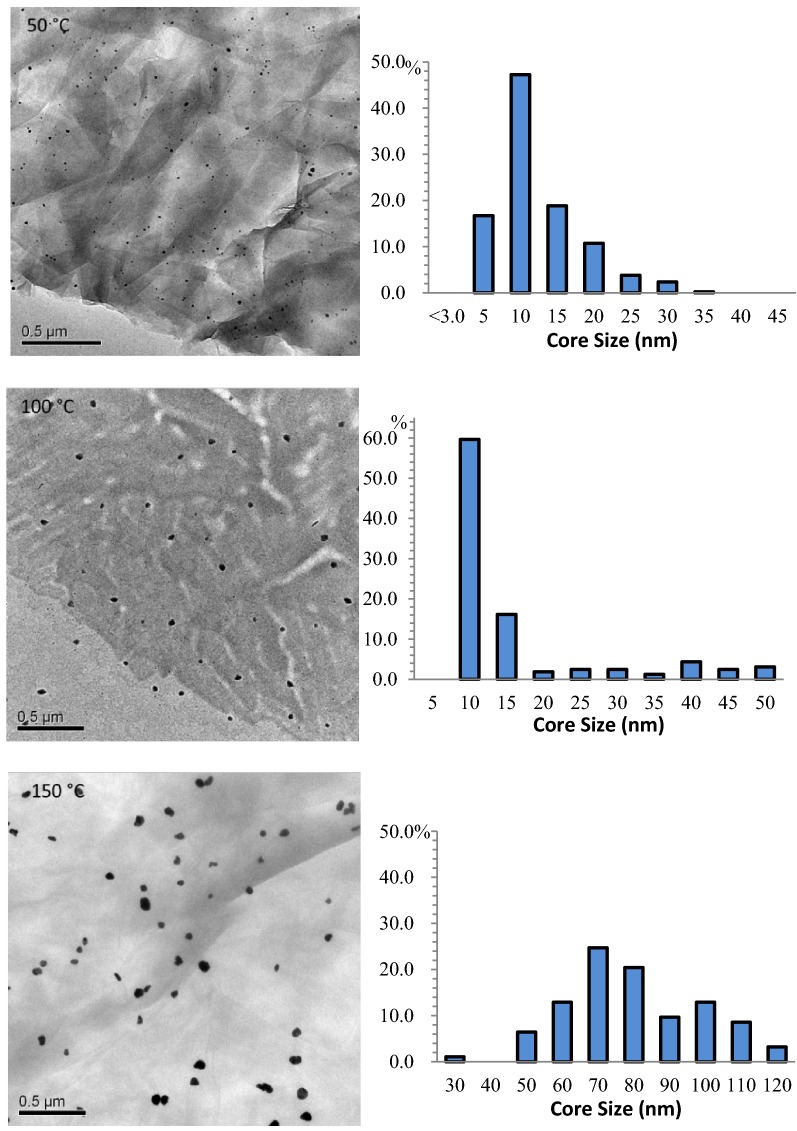
TEM images and histograms of cAuNP_3nm_-GO heated at 50, 100, and 150 °C

To understand the effects of capping ligands on the stability of assembled gold nanoparticles, the heat treatment of cAuNP_3nm_-GO was performed and its results were compared to those of gAuNP_3nm_-GO published in our previous report [24]. UV–vis spectra of the heated cAuNP_3nm_-GO were somewhat different to those of gAuNP_3nm_-GO showing a progressive depression of absorbance intensity. The UV–vis spectra of heat-treated cAuNP_3nm_-GO would only reveal the rapid decrease in the overall absorbance without the appearance of SP or even scattering bands (Fig. 6). This is due to the rapid flocculation of hybrid materials even at 50 °C causing the visible precipitation of cAuNP_3nm_ –GO. TEM images of heated cAuNP_3nm_-GO clearly indicated that cAuNP undergo coarsening to larger particles when heated at 50 °C (Fig. 7). The histogram analyses results shown in Fig. 7 and Table 1 confirmed a very fast increase in the average size of cAuNP to ~ 10, 14, and 68 nm after heat treatments at 50 °C, 100 °C, and 150 °C, respectively. The large increases in average size of cAuNP after heat treatment suggested that the extensive coarsening of cAuNP on GO takes place at lower temperature. The weak binding affinity of CTAB with AuNP most likely causes CTAB to be more easily stripped by the GO and AuNP to diffuse on GO to form aggregates at faster rate.

### Effects of additional thiol functional groups on graphene oxide for coarsening of gold nanoparticles and nanorods (GO vs tGO)

The gAuNP_3nm_ and cAuNR were also adsorbed on thiol-functionalized GO (tGO) to study the effect of additional thiol-gold interaction between nanoparticles and GO during coarsening or reshaping. The tGO was prepared by the well-established method using the coupling of l-cysteine to graphene oxide in the presence of EDC and NHS [22, 30, 32]. The tGO was then mixed with a solution containing gAuNP_3nm_ or cAuNR which resulted in the self-assembly of nanoparticles onto the surface of tGO with the formation of gold-sulfur bonds in addition to all electrostatic interactions unmodified GO provide. TEM and histogram results showed that the gAuNP_3nm_-tGO still have the surface immobilized particles with the average core size near ~ 3 nm (Additional file 1: Figure S6). Any immediate aggregation after the adsorption of gAuNP_3nm_ on tGO was not observed from TEM results.

Additional file 1: Figure S7 shows the UV–vis spectra of gAuNP_3nm_-tGO after heat treatments at temperature ranging from 50 to 200 °C. The intensity of SP bands along with the overall absorbance spectra, which show an exponential decay from lower wavelength to higher wavelength, continuously decreased after heat treatments at the range between 50 and 150 °C. The results were almost identical with the ones observed for gAuNP_3nm_-GO indicating the particles have begun to aggregate and become partially insoluble [24]. The UV–vis spectra of gAuNP_3nm_-tGO heated at 200 °C showed completely different spectral features for the particles heated at high temperature, which resemble the characteristic of localized metallic film-like structures [24, 39–42].

Additional file 1: Figure S8 shows TEM images of heated gAuNP_3nm_-tGO at temperature from 50 to 150 °C. These results showed that the average core size increase occurred the most between 100 and 150 °C as we have seen from the heat treatments of gAuNP_3nm_-GO. However, after heating at 150 °C, TEM image and histogram results showed that the gAuNP_3nm_-tGO had a smaller average core size (5.09 ± 1.97 nm) than gAuNP_3nm_-GO (7.04 ± 3.61 nm). Although the difference is still within the standard deviation of each sample, the result suggests that there might be some stabilizing influence of tGO with the formation of Au–S bonds between tGO and gAuNP (Table 1). The presence of stronger Au–S bonds in addition to other electrostatic interactions would provide more robust interactions between nanoparticles and GO. Thus, with the use of tGO, the immobilized AuNP would be more kinetically trapped and become slightly less susceptible to aggregation and coarsening.

**Fig. 8:**
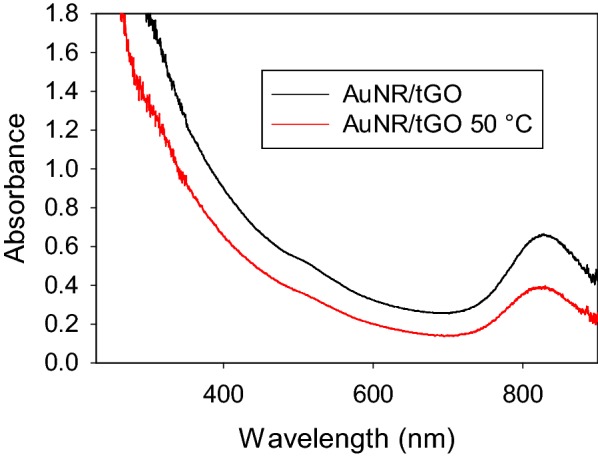
UV-vis spectra of cAuNR-tGO before and after heat treatment at 50 °C

**Fig. 9:**
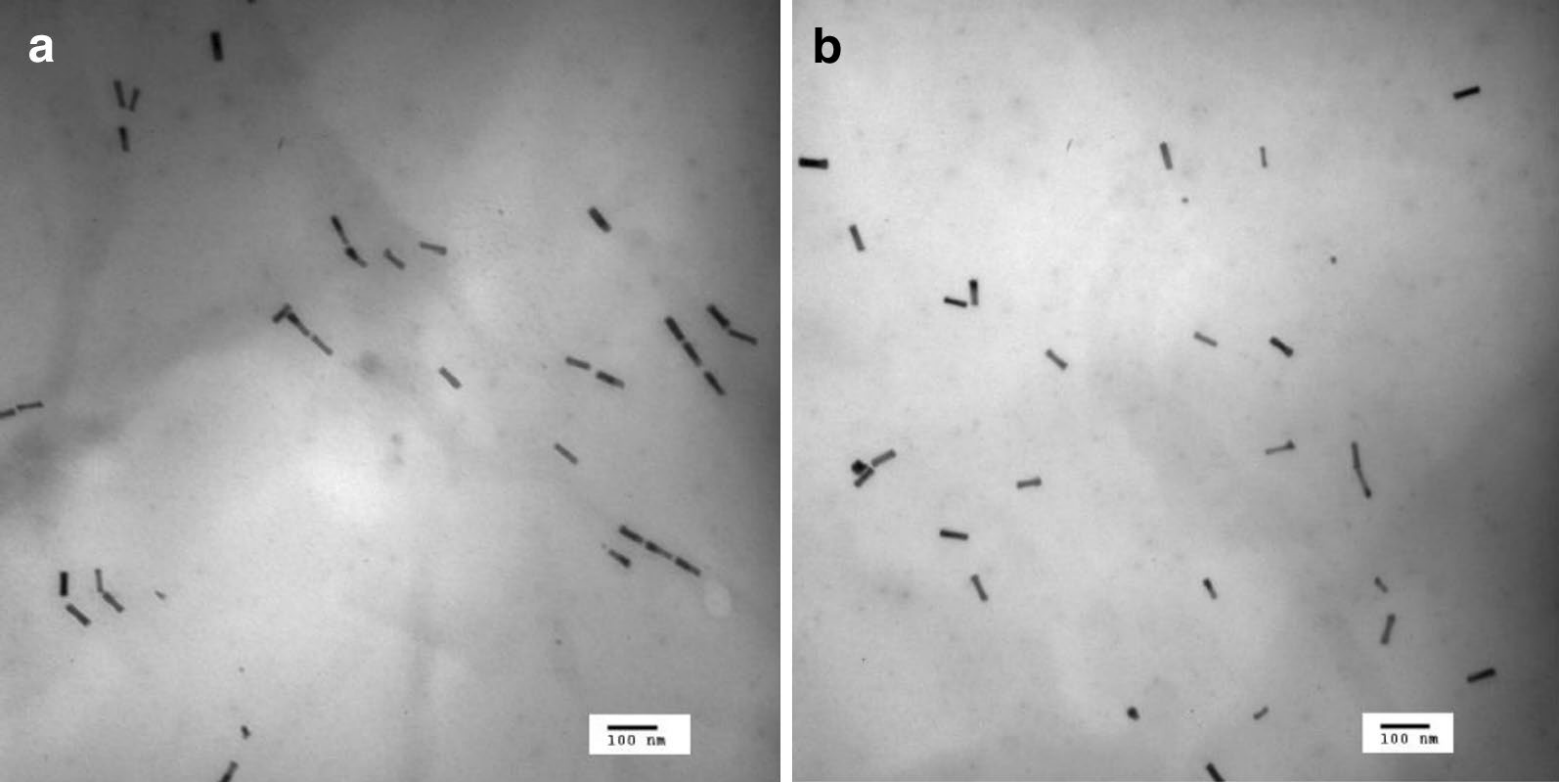
TEM images (scale bars = 100 nm) of **a** cAuNR-tGO and **b** cAuNR-tGO heated at 50 °C

The influence of thiol functional group of tGO on reshaping behavior of cAuNR was also investigated. The cAuNR was adsorbed on the surface of tGO and the heat treatment results at 50 °C were compared to those of cAuNR-GO. Figure 8 shows the UV–vis spectrum of cAuNR-tGO which has the longitudinal SP bands of cAuNR-tGO appear at higher wavelength of ~ 830 nm compared to those of cAuNR and cAuNR-GO. This shift is likely a result from the plasmonic shift caused by the formation of Au–S bonds [6]. TEM image of cAuNR-tGO shown in Fig. 9 confirmed the size and shape of cAuNR remained mostly intact after the adsorption on tGO. UV–vis spectra and TEM image of cAuNR-tGO after heat treatment at 50 °C are also shown in Figs. 8 and 9, respectively. Although the intensity of the longitudinal plasmon band has decreased slightly, the overall spectral feature remains almost constant after heat treatment. This result was different from the previous studies on cAuNR-GO, in which the UV–vis spectra of heated samples at 50 °C showed a complete disappearance of the plasmon band at 780 nm [24]. TEM images also showed that there is little change in nanorod shape when cAuNR-tGO is heated at 50 °C. In comparison, cAuNR-GO underwent a complete reshaping of nanorods to spherical particles after the heat treatment at 50 °C (Table 1) [24]. The results confirmed the presence of stronger interactions between GO and cAuNR and demonstrated dramatically improved stability of AuNR during heat treatments. The heat treatments at temperature 100 °C or higher, however, caused the cAuNR to undergo a complete reshaping to spherical particles.

## Conclusions

In this study, the coarsening behavior of gold nanoparticles on graphene oxide was investigated with the focus on the influence of particle core size, stabilizing ligands, and thiol linkers. For gold nanoparticles, the core size evolution was slightly affected by the size of nanoparticles. Gold nanoparticles smaller than 1.5 nm and large colloidal gold nanoparticles of ~ 10 nm were less stable than gold nanoparticles with ~ 3 nm core, undergoing immediate coarsening and/or aggregation upon adsorption on the surface of graphene oxide. The core size evolution during heat treatments at the temperature ranging from 50 to 200 °C, however, was similar for all three gold nanoparticles with different core sizes. Glutathione was observed to be a more stabilizing ligand than CTAB by having a stronger bond with the gold surface and a greater resistance to being removed from the nanoparticles by graphene oxide. Much greater core size evolution was observed from CTAB-capped gold nanoparticles on graphene oxide. The presence of thiol linkers on graphene oxide allowed for the formation of strong Au–S bonds that immobilized the nanoparticles strongly onto graphene oxide. The improvement in the stability of nanoparticles by Au–S bond formation was even more profound for the gold nanorods. The heat treatments of gold nanorods on graphene oxide at 50 °C resulted in the complete reshaping of nanorods to spherical particles. Under the same condition, the reshaping of gold nanorods on thiol-functionalized graphene oxide was not observed indicating that the formation of Au–S bonds allows the gold nanorod to withstand the thermal energy even after the disruption of CTAB ligands around nanorods. The overall results proved that small modification in the nanoparticle and/or graphene oxide functional groups would have a large influence on intrinsic stability of nanomaterials.

## Additional file

**Additional file 1.** Materials, synthetic methods, instrumentation methods, and TEM images and UV–vis spectra of Au nanoparticles and nanorods.

## Abbreviations

AuNP: gold nanoparticles
AuNR: gold nanorods
GO: graphene oxide
CTAB: cetyltrimethylammonium bromide
tGO: thiol-functionalized graphene oxide
TGA: thermogravimetric analysis
gAuNP: glutathione-capped gold nanoparticles
cAuNP: cetyltrimethylammonium bromide-capped gold nanoparticles
cAuNR: cetyltrimethylammonium bromide-capped gold nanorods
EDC: (1-ethyl-3-(3-dimethylaminopropyl)carbodiimide)
SP: surface plasmon
GS: glutathione
TEM: transmission electron microscopy
SR: alkanethiolate
THF: tetrahydrofuran
