# Supplementary material for: Morphological transformation of gold nanoparticles on graphene oxide: effects of capping ligands and surface interactions
Source: Nano Converg. 2019 Jan 8;6:2. doi: 10.1186/s40580-018-0171-0 (PMC6323066; doi:10.1186/s40580-018-0171-0)
Supplement: Supplementary file 1 — Additional file 1. Materials, synthetic methods, instrumentation methods, and TEM images and UV–vis spectra of Au nanoparticles and nanorods. [file 40580_2018_171_MOESM1_ESM.pdf]

Supplemental Information

Morphological Transformation of Gold Nanoparticles on Graphene Oxide: Effects of Capping Ligands and Surface Interactions

Hanqing Pan, Serena Low, Nisala Weerasuriya, Bingli Wang, and Young-Seok Shon*

Department of Chemistry and Biochemistry, California State University, Long Beach. 1250 Bellflower Blvd. Long Beach, CA 90840. *Email: ys.shon@csulb.edu.

Materials and Methods S-2

Figure S1. TEM images and histograms of (a) $gAuNP_{10nm}$-GO, (b) $gAuNP_{3nm}$-GO, and (c) $gAuNP_{1nm}$-GO. S-6

Figure S2. TEM images and histograms of (a) $cAuNP_{3nm}$-GO and (b) cAuNR-GO**.** S-7

Figure S3. UV-vis spectra of $gAuNP_{10nm}$-GO heated at 50, 100, 150, and 200 °C. S-8

Figure S4. UV-vis spectra of $gAuNP_{1nm}$-GO heated at 50, 100, 150, and 200 °C. S-8

Figure S5. TEM images of $gAuNP_{1nm}$-GO heated at 100, 150, and 200 °C. S-9

Figure S6. TEM image and histogram of $gAuNP_{3nm}$-tGO. S-9

Figure S7. UV-vis spectra of $gAuNP_{3nm}$-tGO heated at 50, 100, 150, and 200 °C. S-10

Figure S8. TEM images of $gAuNP_{3nm}$-tGO heated at 50, 100, and 150 °C. S-10

References S-11

Materials and Methods

Materials. The following materials were purchased from the indicated suppliers and used as received: sodium citrate, silver nitrate, ascorbic acid, and graphene oxide were purchased from Sigma-Aldrich. Methanol, ethanol, glutathione reduced (GSH), and L-cystine were obtained from Fisher Scientific. Hydrogen tetrachloroaurate trihydrate ($HAuCl_4 \cdot 3H_2O$), sodium borohydride ($NaBH_4$), and N-hydroxysuccinimide (NHS) were purchased from Acros. (1-Cetyl)trimethyl ammonium bromide (CTAB) was purchased from Alfa Aesar. 1-Ethyl-3-(3-dimethylaminopropyl)carbodiimide (EDC) was purchased from TCI America. Spectra/Por cellulose ester (CE) dialysis membranes (M. Wt. = 8,000-10,000 Daltons) were purchased from Spectrum Laboratories, Inc. Nanopure water was purified by a Barnstead NANOpure Diamond ion exchange resins purification unit.

Synthesis of 10 nm glutathione-stabilized gold nanoparticles ($gAuNP_{10nm}$). A 1 mM solution of $HAuCl_4$ was prepared by dissolving 98.6 mg of $HAuCl_4$ in 250 mL nanopure water. A 38.8 mM solution of citrate was also prepared by adding 285 mg of citrate in 25 mL nanopure water. A 200 mL aqueous solution of 1 mM $HAuCl_4$ was refluxed for 15 min while stirring vigorously. To the $HAuCl_4$ solution, 20 mL of 38.8 mM citrate was quickly added. The solution was then allowed to reflux for an additional 10 min and cooled to room temperature while stirring. To 10 mL of the gold nanoparticle solution, 99 % nitrogen gas was added to de-aerate the solution for 30 min. A 50 µL sample of 0.5 mol GSH was then added to the solution and the reaction mixture was stirred for 12 h. The sample was dialyzed in nanopure water for 6 h to remove extra free small molecules.

Synthesis of 3 nm glutathione-stabilized gold nanoparticles ($gAuNP_{3nm}$). The synthesis of small glutathione-capped Au nanoparticles was achieved with a slight modification to the

published method.[S1,S2] Briefly, nanoparticles were synthesized by adding the reducing agent ($NaBH_4$) to a gold precursor ($HAuCl_4$) in the presence of a ligand, GSH. A 1 mmol (0.39 g) of $HAuCl_4$ was dissolved in a solution of 30 mL of methanol and 20 mL of water, which resulted in a bright yellow solution. A 1 mmol (0.31 g) of GSH is added to the rapidly stirred gold salt solution. The solution turned light brown and slowly turned colorless within 40 min. A $NaBH_4$ solution, freshly made by dissolving 5 mmol (0.19 g) of $NaBH_4$ in 40 mL of water, was slowly added to the vigorously stirred precursor solution, which caused the solution to immediately turn dark brown due to the formation of gold nanoparticles. The solution was quickly transferred into a water bath that was kept at 50 °C and stirring was continued for 1 h. The resulting solution was evaporated to near dryness on a rotary evaporator. Excess methanol was added to precipitate the particles and wash reaction byproducts and any remaining starting material away. The resulting solution was dialyzed in nanopure water for 72 h.

Synthesis of 1 nm glutathione-stabilized gold nanoparticles (gAuNP$_{1nm}$). To 50 mL of cooled (0 °C) methanol, 98.5 mg (0.25 mmol) of $HAuCl_4{\cdot}3H_2O$ was added and the reaction mixture was stirred until completely dissolved. To this yellow solution, 307.3 mg (1.0 mmol) of GSH was added and the mixture was allowed to stir for an additional 30 min. The yellow solution turned clear within 5 min of the addition of GSH. Following this reaction period, 0.2 M (94.6 mg, 12.5 mL) cooled aqueous solution of $NaBH_4$ was added to the mixture under vigorous stirring. The dark brown reaction mixture was stirred for 1 h and the resulting precipitate was separated by centrifugation. To wash the crude precipitate, several 1 mL increments of methanol were added and then removed, followed by the addition and removal of 1 mL of ethanol. The washed product was allowed to dry in a vacuum oven overnight.

A 36.0 mg of the dried crude product was dissolved in 14 mL of nanopure water and heated to 55 °C with stirring. To the dark brown solution, 112 mg of GSH was added and the reaction was allowed to proceed for 90 min under heating with air slowly bubbling directly into the mixture. After the reaction period, the brownish solution was separated from any unreacted crude product by centrifugation. The brownish solution was purified by adding methanol to induce precipitation, which was then further washed with several increments of 1 mL methanol. The washed product was allowed to dry in a vacuum oven overnight. The $gAuNP_{1nm}$ can also be designated as $Au_{25}(GS)_{18}$, due to the fact that there are exactly 25 gold atoms surrounded by 18 glutathione ligands in each 1 nm AuNP as reported in the referenced publications.[S3,S4]

Synthesis of cetyltrimethylammonium bromide-stabilized gold nanoparticles ($cAuNP_{3nm}$) and nanorods (cAuNR). Gold nanorods were synthesized using the procedure reported by Castellana et al. via a seed-mediated method.[S5] In this method, seed and growth solutions were mixed by introducing a small amount of seed solution to the growth solution, which initiates nanorod growth. Both seed and growth solutions were prepared in separate 20 mL dram vials, which were cleaned with aqua regia, extensively rinsed, and annealed at 150 ºC for 2 h prior to use. The aqueous solution, consisting of 5.0 mL of 0.50 mM $HAuCl_4$ and 5.0 mL of 0.20 M CTAB, was placed in a water bath (27 °C) and stirred at 1000 rpm. The seed particles, which are CTAB-stabilized gold nanoparticles ($cAuNP_{3nm}$), were formed by the rapid addition of 0.60 mL of ice-cold aqueous 0.010 M $NaBH_4$. The resulting seed solution was stirred for 2 min and allowed to age for an additional 10 min prior to use. The growth solution in water was made by mixing 25 µL of 0.040 M $AgNO_3$, 5.0 mL of 0.20 M CTAB, 20 µL of 0.50 M H_2SO_4, 5.0 mL of 1.00 mM $HAuCl_4$, and 70 µL of 0.079 M ascorbic acid (note: the ascorbic acid must be prepared fresh and was added to the mixture last). After the addition of ascorbic acid, the growth solution

was gently mixed until the color changed from dark yellow to colorless. To the growth solution, 12 μL aliquot of seed solution was added. The solution was gently mixed again and incubated at 27 °C. The final growth solution was aged for a minimum of 2 h, in which the color of the solution changed from clear to light peach.

Instrumental Methods. Transmission electron microscopy (TEM) images were taken with a JEOL 1200 EX II electron microscope operating at 90 KeV. Samples dissolved in nanopure water were cast onto carbon-coated copper mesh grids and let dry for at least 24 h before analysis. Gold nanoparticle core sizes were analyzed with Scion Image Beta Release 2.0. UV-vis spectra of nanoparticle, nanorod, and hybrid solutions were obtained with a UV-2450 Shimadzu UV-vis spectrophotometer using quartz cells. Spectra were recorded from 800 to 200 nm (900 to 200 nm for gold nanorod solutions).

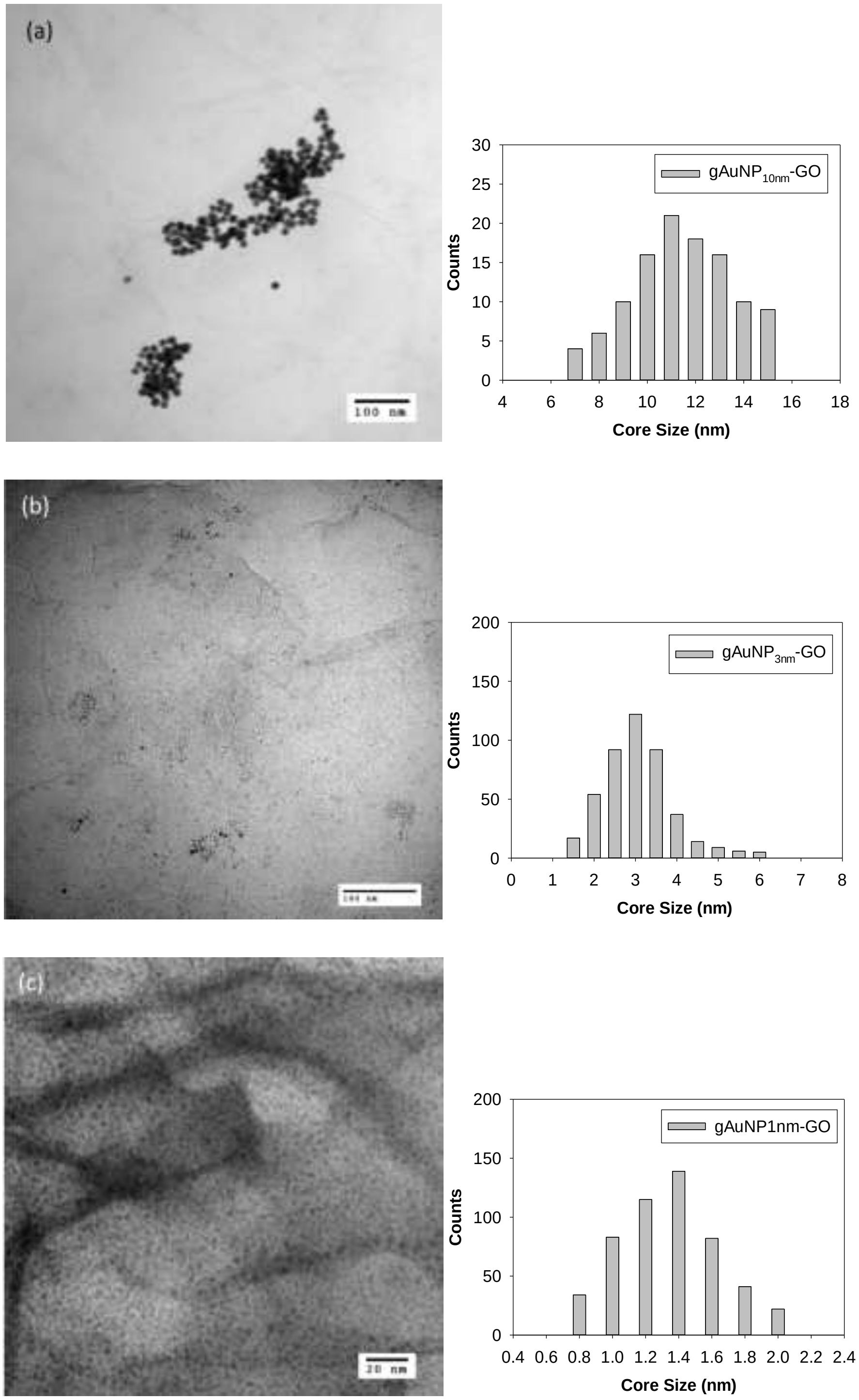

Figure S1. TEM images and histograms of (a) $gAuNP_{10nm}$-GO, (b) $gAuNP_{3nm}$-GO, and (c) $gAuNP_{1nm}$-GO.

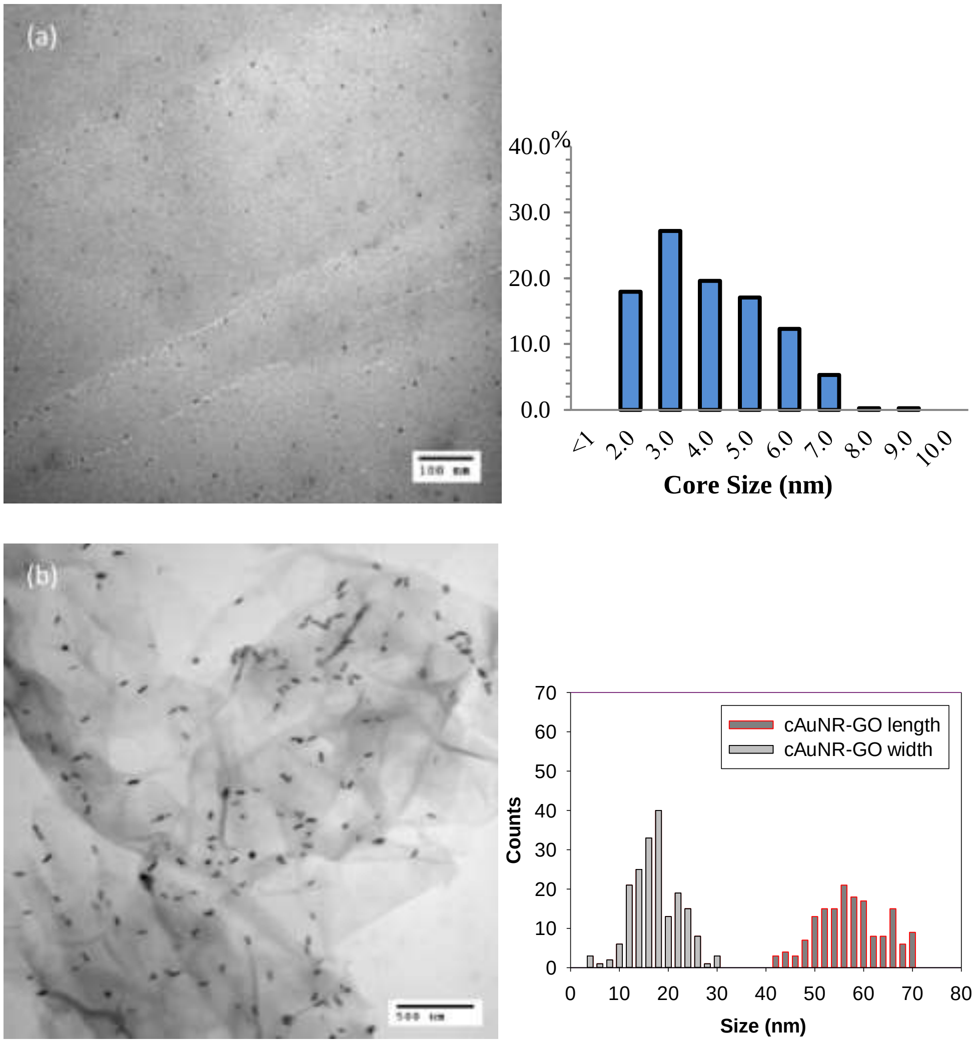

Figure S2. TEM images and histograms of (a) cAuNP$_{3nm}$-GO and (b) cAuNR-GO**.**

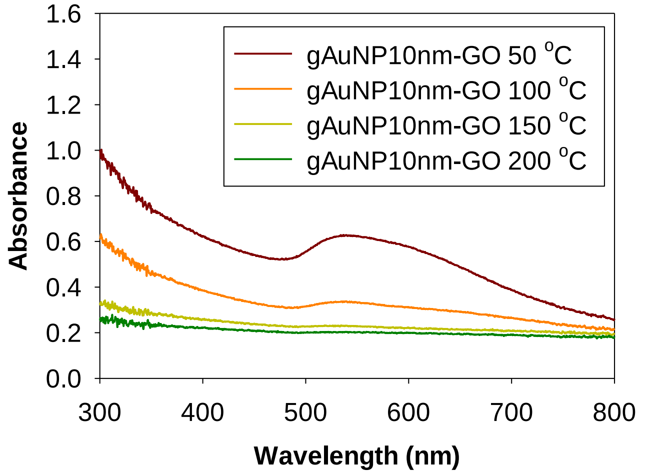

Figure S3. UV-vis spectra of gAuNP$_{10nm}$-GO heated at 50, 100, 150, and 200 °C.

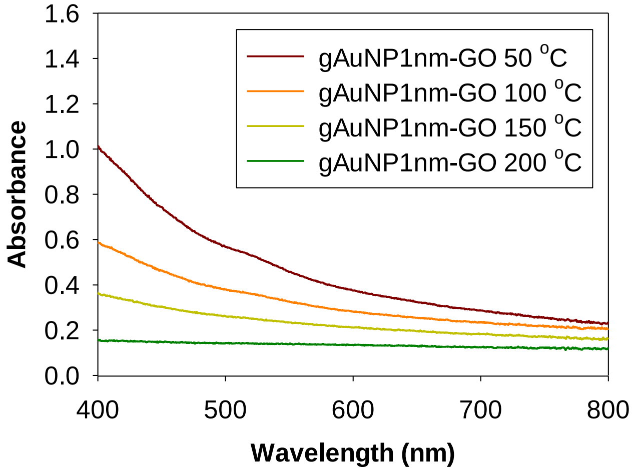

Figure S4. UV-vis spectra of gAuNP$_{1nm}$-GO heated at 50, 100, 150, and 200 °C.

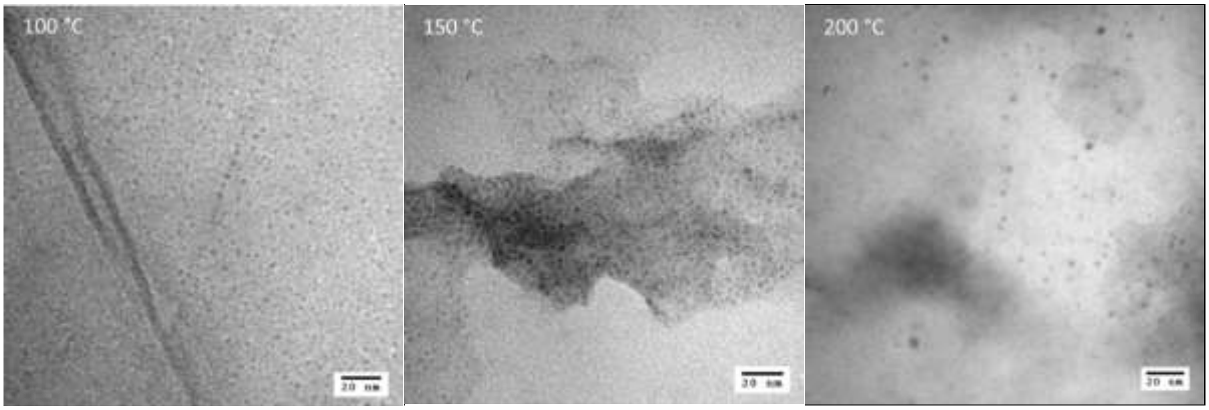

Figure S5. TEM images of gAuNP$_{1nm}$-GO heated at 100, 150, and 200 °C.

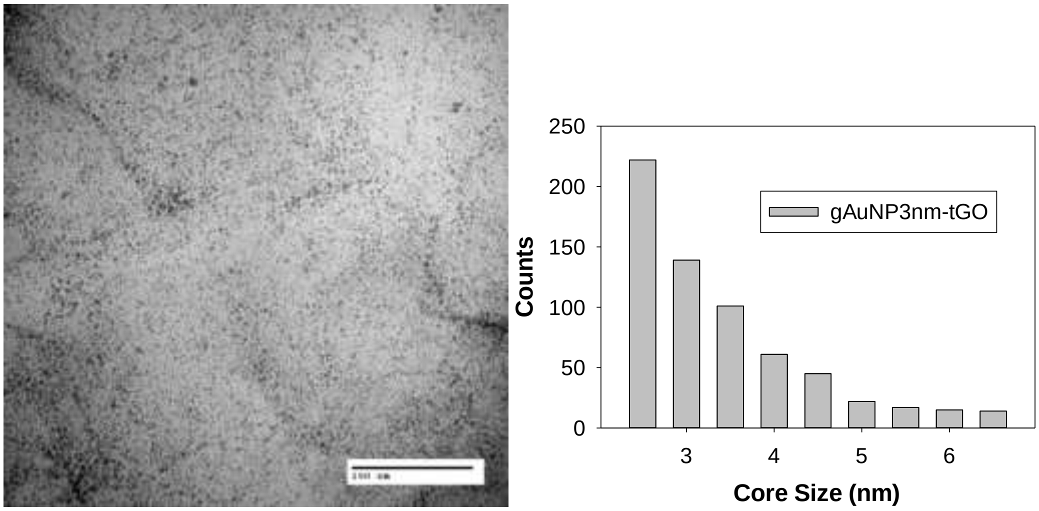

Figure S6. TEM image and histogram of gAuNP$_{3nm}$-tGO.

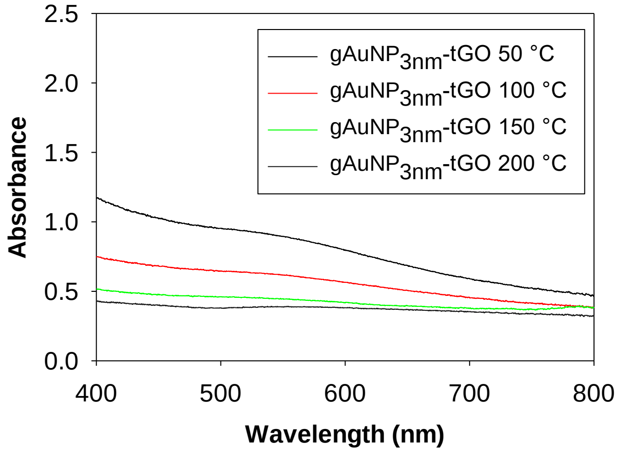

Figure S7. UV-vis spectra of gAuNP$_{3nm}$-tGO heated at 50, 100, 150, and 200 °C.

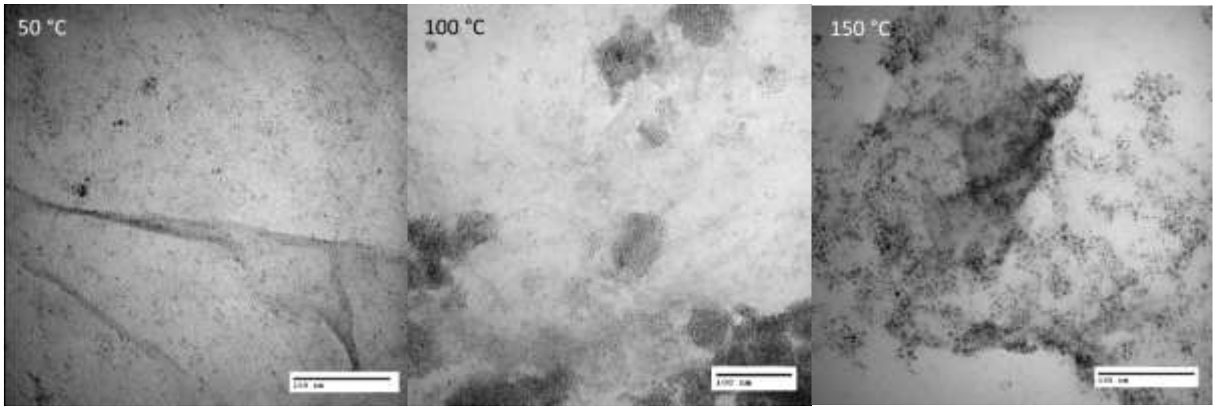

Figure S8. TEM images of gAuNP$_{3nm}$-tGO heated at 50, 100, and 150 °C.

References

S1. KG Stamplecoskie, Y-S Chen, PV Kamat, Excited-state behavior of luminescent glutathione-Protected gold clusters. J. Phys. Chem. C 118, 1370−1376 (2014).

S2. S Deol, N Weerasuriya, Y-S Shon, Stability, cytotoxicity, and cell uptake of water-soluble dendron-conjugated gold nanoparticles with 3, 12, and 17 nm core. J. Mater. Chem. B 3, 6071-6080 (2015).

S3. Z Wu, J Suhan, R Jin, One-pot synthesis of atomically monodisperse, thiol-functionalized Au_{25} nanoclusters. J. Mater. Chem. 19, 622-626 (2009).

S4. S Kumar, R Jin. Water-soluble $Au_{25}(Capt)_{18}$ nanoclusters: Synthesis, thermal stability, and optical properties. Nanoscale 4, 4222-4227 (2012).

S5. ET Castellana, RC Gamez, DH Russell, Label-free biosensing with lipid-functionalized gold nanorods. J. Am. Chem. Soc. 133, 4182−4185 (2011).
